# Single‐oocyte transcriptome analysis reveals aging‐associated effects influenced by life stage and calorie restriction

**DOI:** 10.1111/acel.13428

**Published:** 2021-07-10

**Authors:** Tappei Mishina, Namine Tabata, Tetsutaro Hayashi, Mika Yoshimura, Mana Umeda, Masashi Mori, Yayoi Ikawa, Hiroshi Hamada, Itoshi Nikaido, Tomoya S. Kitajima

**Affiliations:** ^1^ Laboratory for Chromosome Segregation RIKEN Center for Biosystems Dynamics Research (BDR) Kobe Japan; ^2^ Graduate School of Biostudies Kyoto University Kyoto Japan; ^3^ Laboratory for Bioinformatics Research RIKEN Center for Biosystems Dynamics Research (BDR) Kobe Japan; ^4^ Laboratory for Organismal Patterning RIKEN Center for Biosystems Dynamics Research (BDR) Kobe Japan; ^5^ Department of Functional Genome Informatics, Division of Medical Genomics, Medical Research Institute Tokyo Medical and Dental University (TMDU) Bunkyo Japan; ^6^ Master's/Doctoral Program in Life Science Innovation (Bioinformatics), Degree Programs in Systems and Information Engineering, Graduate School of Science and Technology University of Tsukuba Tsukuba Japan

**Keywords:** aging, chromosome segregation, cohesin, oocyte, transcriptome

## Abstract

Chromosome segregation errors in oocytes lead to the production of aneuploid eggs, which are the leading cause of pregnancy loss and of several congenital diseases such as Down syndrome. The frequency of chromosome segregation errors in oocytes increases with maternal age, especially at a late stage of reproductive life. How aging at various life stages affects oocytes differently remains poorly understood. In this study, we describe aging‐associated changes in the transcriptome profile of mouse oocytes throughout reproductive life. Our single‐oocyte comprehensive RNA sequencing using RamDA‐seq revealed that oocytes undergo transcriptome changes at a late reproductive stage, whereas their surrounding cumulus cells exhibit transcriptome changes at an earlier stage. Calorie restriction, a paradigm that reportedly prevents aging‐associated egg aneuploidy, promotes a transcriptome shift in oocytes with the up‐regulation of genes involved in chromosome segregation. This shift is accompanied by the improved maintenance of chromosomal cohesin, the loss of which is a hallmark of oocyte aging and causes chromosome segregation errors. These findings have implications for understanding how oocytes undergo aging‐associated functional decline throughout their reproductive life in a context‐dependent manner.

## Graphical abstract

This study reports single‐oocyte transcriptome analysis with RamDA‐seq during aging and calorie restriction in mice. Oocytes undergo aging‐associated transcriptome changes at a late reproductive stage, when chromosome segregation errors are pronouncedly increased, while their surrounding cumulus cells exhibit gradual transcriptome changes even at earlier life stages. Calorie restriction promotes a transcriptome shift in oocytes, accompanied with improved maintenance of chromosomal cohesin.

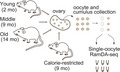

## 1 INTRODUCTION

Fertility in human females declines with age. Until menopause, which marks the end of the reproductive life by ceasing the ovulation cycle, the rates of infertility and pregnancy loss increase with age. One of the major contributors to these increases is egg aneuploidy. Aneuploidy is found in ~35% of spontaneous abortions, and most of the aneuploidies are derived from eggs (Nagaoka et al., 2012). In fertility clinics, 30%–70% of human eggs are found to be aneuploid, and the rate of aneuploidy increases with age (Fragouli et al., 2011; Gabriel et al., 2011; Geraedts et al., 2011). Aneuploid eggs are produced by chromosome segregation errors during meiosis in oocytes. How maternal aging leads to elevated rates of chromosome segregation errors in oocytes remains incompletely understood (Chiang et al., 2012; Herbert et al., 2015; Jones & Lane, 2013; Mihajlović & FitzHarris, 2018; Nagaoka et al., 2012; Webster & Schuh, 2017).

Aging impacts the transcriptome landscape of cells, and the effects depend on the type of cells. In ovarian follicles, oocytes communicate with their surrounding cumulus cells through gap junctions, which have critical roles in oocyte development and function (Kidder & Mhawi, 2002; Su et al., 2009). Although previous studies have described aging‐associated changes in the transcriptome of oocytes, whether those changes are coordinated with their surrounding cumulus cells remains unknown. Previous studies used microarray approaches, which identified differential patterns of gene expression between the oocytes of young and old mice (Hamatani et al., 2004; Pan et al., 2008). Recently, single‐oocyte RNA sequencing described transcriptome differences between mouse oocytes at 8 months old and at 1 month old (Zhang et al., 2019). However, chronological changes in the transcriptome landscape of single oocytes up to a late reproductive stage, at which aging‐associated chromosome segregation errors are pronouncedly increased (e.g., 7–25% at ~15 months old in BDF1 mice (Pan et al., 2008; Chiang et al., 2010; Sakakibara et al., 2015)), remain unknown.

Reproduction can be affected by nutrition and by metabolic state. Calorie restriction (CR), which retards aging‐associated functional declines in many experimental models (Benayoun et al., 2015; Guarente, 2008; Zhang et al., 2020), prevents aging‐associated increases in egg aneuploidy in mouse oocytes (Selesniemi et al., 2011). CR prevents aging‐associated increases in spindle abnormalities in oocytes (Selesniemi et al., 2011), which may partly explain the CR‐dependent prevention of aneuploidy. However, the vast majority of chromosome segregation errors in oocytes of naturally aged mice are preceded by precocious chromosome separation (Sakakibara et al., 2015). This defect is at least partly due to the aging‐associated, irreversible reduction of chromosomal cohesin, a protein complex that mediates chromosome cohesion (Burkhardt et al., 2016; Chiang et al., 2010; Lister et al., 2010). Thus, the CR‐dependent prevention of egg aneuploidy may be mediated by the suppression of aging‐associated reduction of chromosomal cohesin. Whether dietary conditions influence the aging‐associated reduction of chromosomal cohesin in oocytes, and if so, whether the influences are associated with transcriptome changes in oocytes and their surrounding cumulus cells, remains unknown.

In this study, we characterized the transcriptome datasets of single oocytes and their surrounding cumulus cells at different life stages up to a late reproductive stage in mice. The results show that oocytes exhibit dramatic changes in their transcriptome profiles at a late reproductive stage, whereas their surrounding cumulus cells undergo changes at an earlier stage. CR promoted a transcriptomic shift in oocytes, with the up‐regulation of genes involved in chromosome segregation. At the protein level, CR attenuated the aging‐associated reduction of chromosomal cohesin. Thus, aging‐associated effects on oocytes depend on life stages and can be modified by dietary conditions.

## 2 RESULTS

### 2.1 Transcriptome profiling of single oocytes and their surrounding cumulus cells from mice at different life stages

**FIGURE 1:**
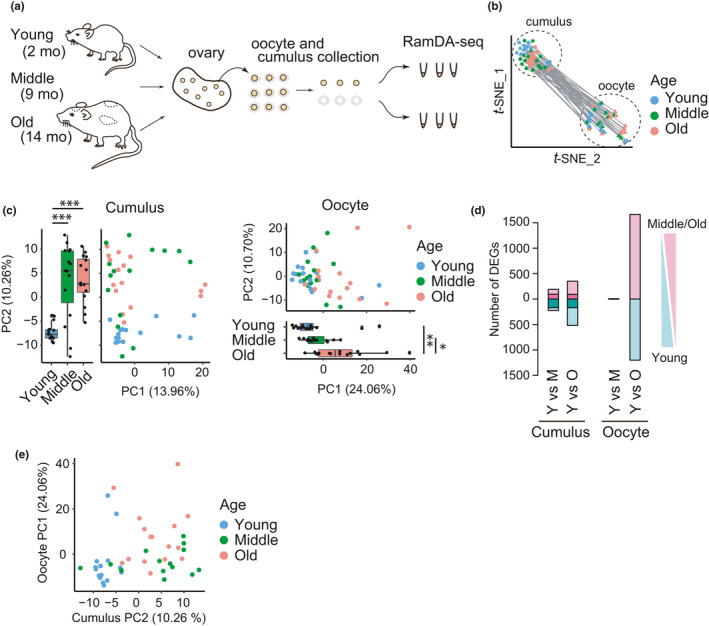
Transcriptome profiling of single oocytes and their surrounding cumulus cells from mice at different life stages. (a) Design overview of RNA sequencing with RamDA‐seq for single oocytes and their surrounding cumulus cells from mice at different ages. (b) *t*‐SNE plot showing the distribution of single oocytes and their surrounding cumulus cells. Circles and triangles represent cumulus cells and oocytes, respectively. The pairs of oocytes and their surrounding cumulus cells are connected with a line. (c) PCA plot using all the expressed genes showing an aging‐associated distribution. (left) cumulus cells and (right) oocytes. Pairwise t test with holm correction was performed (Cumulus: Y vs M, *p *= 1.1e‐5; Y vs O, *p *= 6.2e‐6; M vs O, *p *= 0.84; Oocyte: Y vs M, *p *= 0.755; Y vs O, *p *= 0.0049; M vs O, *p *= 0.0107). (d) Histogram showing the number of up‐regulated or down‐regulated DEGs between young and middle or old groups in cumulus cells (left) and oocytes (right). (e) The profile of oocytes is not predictable from that of cumulus cells. Plot showing the relationship between eigen values of aging‐associated PC axes from oocytes and that from their corresponding cumulus cells

To investigate aging‐associated changes in the transcriptomic landscape of single oocytes and their surrounding cumulus cells, we obtained oocyte–cumulus complexes of fully grown follicles from young (2 months), from middle (9 months), and from old (14 months) BDF1 mice after hyperovulation (Figure 1a). In this strain of mice, aging‐associated chromosome segregation errors are observed around 15 months old (Chiang et al., 2010; Pan et al., 2008; Sakakibara et al., 2015). Consistently, our whole‐mount immunostaining followed by 3D confocal imaging of oocytes showed that chromosome abnormalities at metaphase II (including premature separation of chromatids and abnormal numbers of kinetochores) increased during aging, gradually from 2 to 9 months and pronouncedly from 9 to 14 months (Figure S1a and S1b). Thus, the old mice analyzed in this study are suitable for exploring transcriptome changes associated with age‐related errors. Individual oocyte–cumulus complexes were separated into an oocyte and a pool of its surrounding cumulus cells, which were subjected to RamDA‐seq, a technique for full‐length total RNA sequencing (Hayashi et al., 2018) (Figure 1a). After sample filtration, the high‐quality transcriptomes of 46 single oocytes and their surrounding cumulus cells, which were collected from 2 young, 4 middle, and 3 old mice, were retained for subsequent analyses. Using the datasets of young, middle, and old mice, we performed *t*‐distributed stochastic neighbor embedding (*t*‐SNE) dimensionality analysis. We detected two distinct cell clusters corresponding to oocytes and cumulus cells (Figure 1b, S2a and S2b), which indicated the robustness of our cell isolation technique. Moreover, oocytes collected from old mice exhibited normal expression levels of *Stella*, *Oct3*/*4*, and *Nyfa*, marker genes for fully grown oocytes (Zuccotti et al., 2008) (Figure S2c), which confirmed that oocytes and cumulus cells were collected from follicles at the same developmental stage.

### 2.2 Oocytes undergo aging‐associated transcriptome changes with increased heterogeneity at a late stage of reproductive life

We then separately analyzed datasets from oocytes and from their surrounding cumulus cells using principal component analysis (PCA) (Figure 1c). PCA of cumulus cells detected significant changes from young to middle mice along the PC2 axis, while no significant difference was detected from middle to old mice (Figure 1c, Cumulus). In contrast, PCA of oocytes detected no significant changes from young to middle mice along the PC1 axis that showed a significant difference from middle to old mice (Figure 1c, Oocyte). These results suggest that aging‐associated global changes in the transcriptome occur during relatively early life stages in cumulus cells, while oocytes largely maintain their transcriptome until they undergo global changes at a later life stage. Consistent with this idea, we detected a substantial number of differentially expressed genes (DEGs) in cumulus cells between young and middle mice (Middle down‐regulated: n = 225; up‐regulated: n = 190), as well as between young and old mice (Aged down‐regulated: n = 520; up‐regulated: n = 350) (Figure 1d, Cumulus; Figure S3a). In contrast, the number of DEGs in oocytes was small between young and middle mice (Middle down‐regulated: n = 3; Middle up‐regulated: n = 10), but it dramatically increased between young and old mice (Aged down‐regulated: n = 1,201; up‐regulated: n = 1,663) (Figure 1d, Oocyte; Figure S3b). Pairwise analysis of oocytes and their surrounding cumulus cells showed that the PC2 values of oocytes were not correlated with the PC1 values of cumulus cells (Figure 1e), suggesting that aging‐associated transcriptome changes are poorly coordinated between the oocyte and its surrounding cumulus cells in individual follicles. These results were largely reproduced by experiments with a second set of samples, which included oocytes and their cumulus cells from mice at 2, 4, 9, and 14 months old (Figure S3c–e). We also examined samples from 2‐month‐old mice without hyperovulation, and found that hyperovulation had a substantial impact on the transcriptome of cumulus cells but not on that of oocytes (Figure S3f). Collectively, these results indicate that oocytes undergo aging‐associated transcriptome changes at a late stage of reproductive life, in an uncoordinated fashion with their surrounding cumulus cells.

### 2.3 Distinct characteristics of aging‐associated genes between oocytes and cumulus cells

**FIGURE 2:**
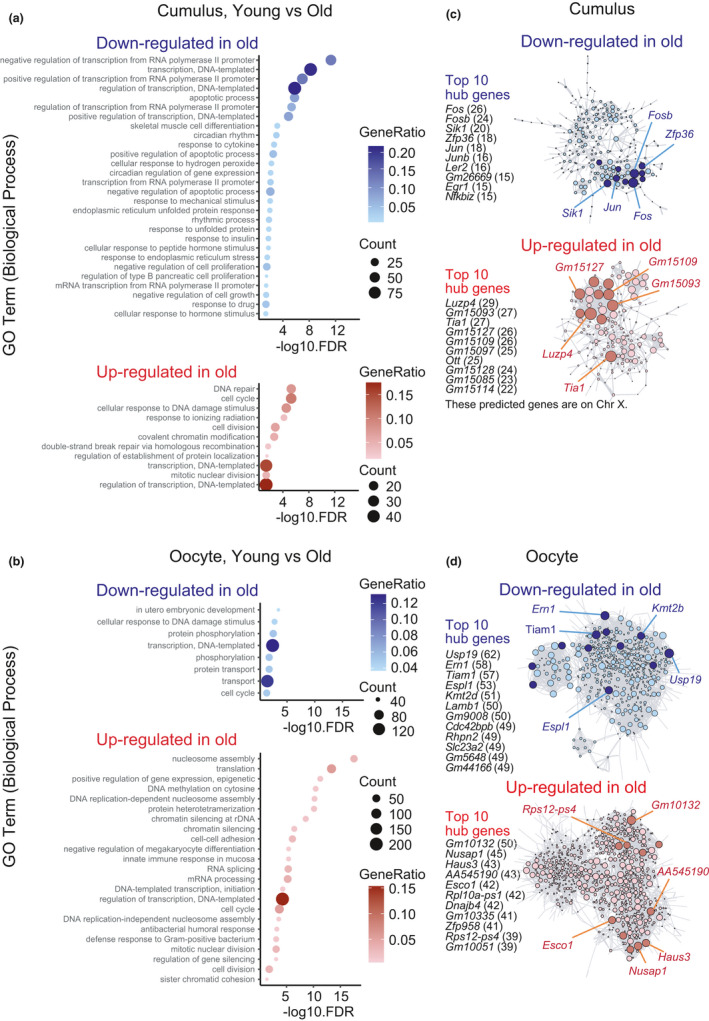
Distinct characteristics of aging‐associated genes between oocytes and cumulus cells. (a) Representative GO terms of down‐regulated (top) and up‐regulated (bottom) enrichment between young and old cumulus cells. (b) Representative GO terms of down‐regulated (top) and up‐regulated (bottom) enrichment between young and old oocytes. (c) Regulatory networks visualizing potential key transcriptional regulators in cumulus cells associated with aging. Down‐regulated and up‐regulated top 10 nodes are colored dark blue and dark red, respectively. (d) Regulatory networks visualizing potential key transcriptional regulators in oocytes associated with aging. Down‐regulated and up‐regulated top 10 nodes were colored in dark blue and dark red, respectively. The threshold used for regulator‐target connection in oocytes was lower than in cumulus cells

Gene ontology (GO) analysis of DEGs revealed distinct characteristics of aging‐associated changes in transcriptomes between oocytes and cumulus cells. Comparison of the datasets of old mice to those of young mice showed that in cumulus cells, genes involved in transcription regulation were predominantly down‐regulated with aging, while genes involved in DNA damage/repair and cell cycle regulation were up‐regulated (Figure 2a). In contrast, in oocytes, genes involved in DNA damage response and *in utero* embryonic development were down‐regulated with aging, while genes related to chromatin regulation, such as nucleosome assembly and epigenetic modification, were markedly up‐regulated (Figure 2b). Moreover, genes involved in cell cycle regulation were aberrantly regulated in the oocytes of old mice, consistent with previous reports (Hamatani et al., 2004; Pan et al., 2008). DEGs detected in the oocytes of middle mice included genes involved in oxidation–reduction processes and steroid hormone synthesis (Figure S3g).

To identify regulators of the aging‐associated changes in gene expression, we constructed transcriptional regulatory networks of core transcriptional regulators and their target genes (Huynh‐Thu et al., 2010). That analysis revealed a core hub of aging‐up‐regulated genes, including *Luzp4*, *Gm15093*, *Tia1*, *Gm15127*, and *Gm15109*, and a core hub of aging‐down‐regulated genes, including *Fos*, *Fosb*, *Sik1*, *Zfp36*, and Jun, in cumulus cells (Figure 2c). However, in oocytes, the connections of the network were relatively weak, although lowering the detection thresholds allowed us to identify a potential hub that regulates the expression of genes associated with aging (Figure 2d). Together, our results reveal the regulatory networks of gene expression programs underlying the aging of oocytes and their surrounding cumulus cells.

### 2.4 Pronounced effects of calorie restriction on transcriptome profiles in oocytes

**FIGURE 3:**
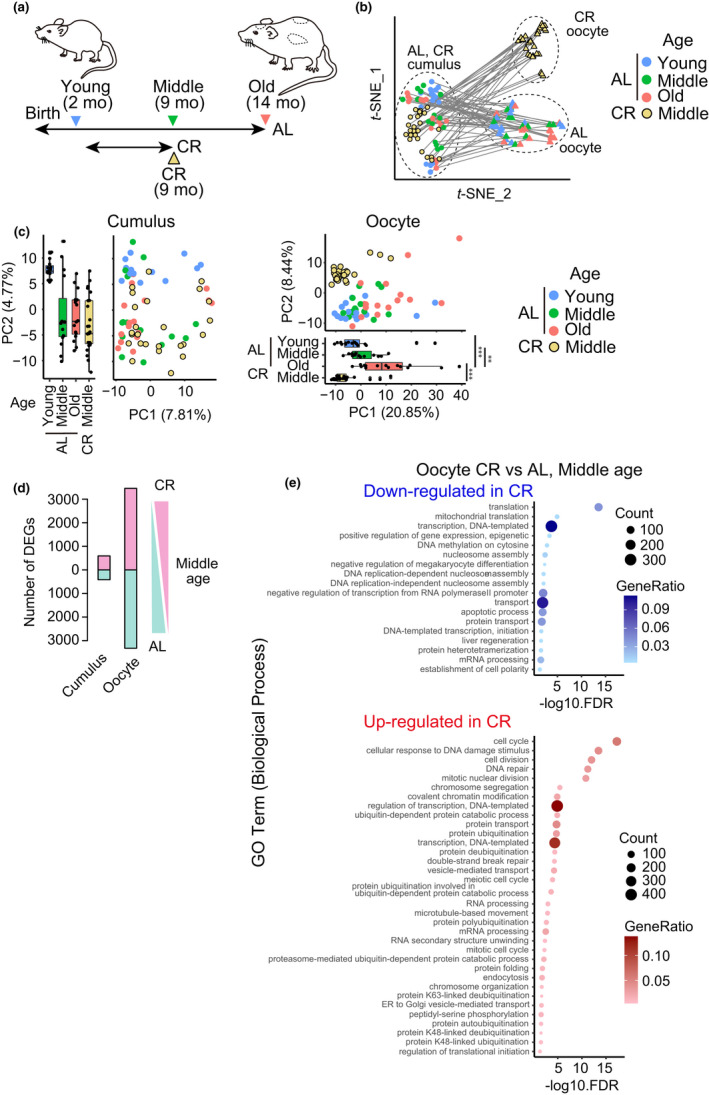
Pronounced effects of CR on transcriptome profiles in oocytes. (a) Design overview of CR. Note that the AL datasets used in this figure are identical to those used in Figure 1. (b) *t*‐SNE plot showing the distribution of single oocytes and their surrounding cumulus cells. Circles and triangles represent cumulus cells and oocytes, respectively. The pairs of oocytes and their surrounding cumulus cells are connected with a line. (c) PCA plot of all expressed genes in cumulus cells (left) and in oocytes (right), respectively. **p < 0.01 and ***p < 0.001 (pairwise t test with holm correction). (d) The number of DEGs between CR and AL in oocytes at middle age. (e) Representative GO terms of down‐regulated (top) and up‐regulated (bottom) enrichment between CR and AL oocytes

We next analyzed whether transcriptome profiles in oocytes and their surrounding cumulus cells are affected by CR. We used an adult‐onset 40% feeding regimen that was reported to prevent aging‐associated oocyte aneuploidy (Selesniemi et al., 2011; Turturro et al., 1999), and obtained oocytes and their surrounding cumulus cells from 9‐month‐old (middle) mice (n = 27 oocytes from 3 mice) (Figure 3a). *t*‐SNE analysis demonstrated that oocytes from CR mice formed a cluster that was distinct from oocytes from ad libitum‐fed (AL) mice at any age, while the cluster of cumulus cells from CR mice was positioned adjacent to that of cumulus cells from AL mice (Figure 3b). While PCA of cumulus cells showed no clear separation between CR‐middle and AL‐middle mice along an axis that appeared to represent aging‐associated effects (Figure 3c, Cumulus, along the PC2 axis), CR tended to shift oocytes in the opposite direction to aging‐associated effects along the PC1 axis (Figure 3c, Oocyte, compare CR‐middle with AL‐middle). Moreover, oocytes showed a substantially greater number of DEGs between CR‐middle and AL‐middle mice (CR down‐regulated: n = 3,488, up‐regulated: n = 3,630), compared to cumulus cells (CR down‐regulated: n = 416, up‐regulated: n = 593; Figure 3d). These results suggest that oocytes have a greater transcriptome response to CR than cumulus cells.

The GO analysis revealed that CR preferentially down‐regulated genes involved in translation, mitochondrial translation, and transcription in oocytes (Figure 3e). Interestingly, GO terms for CR‐up‐regulated genes in oocytes included cell cycle/division, DNA damage/repair, nuclear division, and chromosome segregation (Figure 3e), while their surrounding cumulus cells had up‐regulated genes involved in DNA replication and cell cycle (Figure S4a). Up‐regulated genes involved in chromosome segregation in oocytes included those encoding proteins regulating kinetochore‐microtubule attachment (Knl1, CENP‐E/H/W/F, Spc25, Mis12, Ndc80, Ska3, Bub1, HJURP, and Meikin), spindle assembly (Kif2c, Kif11, and Nek2), and chromosome cohesion (Stag1/SA1, Sgo1, Rad21, and Esco2). Construction of transcriptional regulatory networks suggested core regulators of CR‐associated changes in cumulus cells and oocytes (Figure S4b and S4c). Overall, our analysis demonstrates that CR substantially affects oocytes with transcriptome changes including the elevated transcription of genes involved in chromosome segregation.

### 2.5 Calorie restriction attenuates aging‐associated reduction of chromosomal cohesin

**FIGURE 4:**
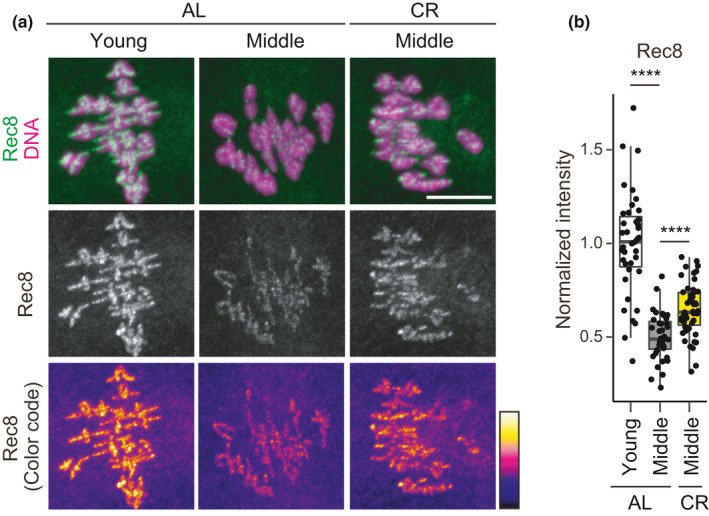
CR attenuates the aging‐associated reduction of chromosomal cohesin. (a) Oocytes were stained for Rec8 (green) and DNA (Hoechst33342, magenta). Z‐projection images are shown. Scale bar, 10 μm. (b) Chromosomal Rec8 signals were quantified. Wilcoxon rank sum test with holm correction was performed (AL‐young v.s. AL‐middle, *p *= 3.5e‐14; AL‐middle v.s. CR‐middle, 2.8e‐16)

The finding of CR‐dependent up‐regulation of genes involved in chromosome segregation, including chromosome cohesion, prompted us to explore the possibility that CR influences chromosomal cohesin, the loss of which with aging is best described as a molecular hallmark of oocytes undergoing chromosome segregation errors (Chiang et al., 2010; Lister et al., 2010). To sensitively detect chromosomal cohesin, we microinjected antibodies against Rec8, a meiotic cohesin subunit essential for chromosome cohesion, into oocytes, fixed them for immunostaining (Lee et al., 2011), and then analyzed by an automated pipeline for measuring signal intensities on chromosomes (Figure 4a). This objective quantification demonstrated that levels of chromosomal Rec8 in middle oocytes were significantly reduced compared to young oocytes. These observations confirmed the findings of previous studies (Chiang et al., 2011; Lister et al., 2010) and demonstrate that the aging‐associated reduction of chromosomal cohesin is initiated at relatively early life stages. Notably, the levels of chromosomal Rec8 in CR oocytes were significantly higher than those in AL oocytes at middle age (Figure 4a,b). These results show that CR attenuates the aging‐associated reduction of chromosomal cohesin in oocytes.

## 3 DISCUSSION

The results of this study indicate that aging‐associated effects on ovarian follicles depend on cell type, life stage, and dietary conditions. Whereas cumulus cells undergo aging‐associated transcriptome changes during early stages of reproductive life, oocytes undergo transcriptome changes at later stages of reproductive life. CR impacts the transcriptome of oocytes to a greater extent than that of cumulus cells. CR up‐regulates genes involved in chromosome segregation, which is associated with the improved maintenance of chromosomal cohesin.

Our results indicate that oocytes and their surrounding cumulus cells undergo different aging‐associated changes in transcriptome profiles. The transcriptome profile of oocytes is relatively stable against aging during the early stages of reproductive life, compared to the transcriptome profile of cumulus cells. In the late stages of reproductive life, however, oocytes undergo global changes in the transcriptome. These late‐onset changes appear to be associated with a sharp increase in the rate of aneuploidy in eggs during the late stages of reproductive life, although whether this is a causal relationship is unknown. Our single‐oocyte approach allowed us to perform a pairwise analysis of oocytes and their surrounding cumulus cells, which suggested that the degree of aging of individual oocytes at the transcriptome level is unpredictable from the transcriptome of their surrounding cumulus cells.

Our results demonstrate that CR substantially impacts oocytes. CR elevated the expression of genes involved in chromosome segregation and attenuated aging‐associated reduction of chromosomal cohesin. Whether these effects contribute to the CR‐dependent prevention of egg aneuploidy, reported previously (Selesniemi et al., 2011), remains unknown. Our study did not directly address CR‐dependent prevention of egg aneuploidy, for which readers are referred to (Selesniemi et al., 2011). The connection between nutrition and oocyte quality is observed in several animal models. In *Drosophila*, a proper nutrient state during oogenesis is critical for the establishment of the unique quiescent state of oocytes (Sieber et al., 2016). In mammals, primordial oocytes are retained in a quiescent state that is prolonged with aging, and proteostatic regulation during the primordial stage is critical for the long‐term maintenance of chromosomal cohesin (Burkhardt et al., 2016; Reichmann et al., 2020). Mouse oocytes have a functional insulin signaling cascade (Acevedo et al., 2007), which may be involved in the transcriptional response to CR. Insulin signaling is a key regulator of the function of mitochondria, which are the primary source of reactive oxygen species (ROS) (Guarente, 2008). In *Drosophila*, levels of the ROS scavenger superoxide dismutase (SOD) are critical for the long‐term maintenance of chromosome cohesion in oocytes (Marquardt et al., 2016; Perkins et al., 2016). Thus, the modulation of oxidative stress is a potential mechanism that might be responsible for the CR‐dependent attenuation of aging‐associated reduction of chromosomal cohesin in oocytes. Alternatively, the up‐regulation of genes involved in the maintenance of chromosome cohesion may also be involved in the CR‐dependent effects on chromosomal cohesin.

Overall, this study reveals life stage‐dependent changes in the transcriptome of oocytes and the influence of CR. Further investigation of molecular links from nutrition signals to aging‐associated transcriptome dysregulation and protein deterioration in oocytes will be important for understanding the maintenance of female reproductive lifespan.

## 4 EXPERIMENTAL PROCEDURES

### 4.1 Mice

All animal experiments were approved by the Institutional Animal Care and Use Committee at RIKEN Kobe Branch (IACUC). B6D2F1 (C57BL/6 × DBA/2) female mice were used to obtain oocytes. For CR, we used an adult‐onset 40% feeding regimen as previously described (Selesniemi et al., 2011; Turturro et al., 1999). Mice were housed individually and fed once a day with a restricted amount of MFG (Oriental Kobo). CR was initiated in a stepwise manner: 10% CR at 14 weeks (3.5 months), 25% CR at 15 weeks, and 40% CR at 16 weeks (4 months). The 40% CR was continued until 9 months of age. Water was provided AL. Effectiveness of the CR protocol was confirmed by monitoring body weight.

### 4.2 Oocyte collection

Mice were injected for hyperovulation with equine chorionic gonadotropin (eCG, ASKA Pharmaceutical) or CARD HyperOva (KYUDO). Ovaries were collected 48 h after injection and put into M2 medium containing 200 nM 3‐isobutyl‐1‐methyl‐xanthine (IBMX, Sigma) at 37°C. Fully grown follicles were dissected to obtain cumulus–oocyte complexes. From each follicle, an oocyte and a pool of its surrounding cumulus cells were isolated with gentle pipetting. Oocytes at the GV stage and their surrounding cumulus cells were retained for further experiments. After washed in 0.1% PBA in PBS, individual oocytes and their surrounding cumulus cells were manually collected in 10 μl cell lysis buffer [10 U RNasein plus (Promega), 10% RealTime ready Cell Lysis Buffer (Roche), 0.3% NP40 (Thermo Fisher Scientific), and RNase‐free water (TAKARA)] in a 0.2 mL single tube. The collected cell samples were lysed and stored at −80℃ until downstream preparation.

### 4.3 RamDA‐seq

The sample single‐tubes were assembled into a 96‐well plate format by a tube tray and holder (Nalge Nunc). The cell lysates were dissolved at 10°C and were agitated for 2 min at 2,000 rpm using a ThermoMixer C at 4°C. The dissolved cell lysates were denatured at 70°C for 90 s. For oocyte samples, 10 µl genomic DNA digestion mix (0.5× PrimeScript Buffer, 2 U DNase I Amplification Grade, 1 µl 500,000‐fold diluted ERCC RNA Spike‐In Mix I (Thermo Fisher Scientific) in RNase‐free water) was added to 10 µl of each denatured sample, which was then incubated at 30°C for 5 min and held at 4°C. Two µl of the digested products was dispensed into 96‐well PCR plates, and 1 µl reverse transcription with random displacement amplification (RT‐RamDA) mix (2.5× PrimeScript Buffer, 0.6 pmol oligo(dT)18 (Thermo Fisher Scientific), 8 pmol 1st‐NSRs 100 ng T4 gene 32 protein (New England Biolabs), and 3× PrimeScript enzyme mix (TAKARA) in RNase‐free water) were added. The samples were incubated at 25°C for 10 min, at 30°C for 10 min, at 37°C for 30 min, at 50°C for 5 min, and then at 94°C for 5 min. For cumulus cell samples, the denatured cell lysate samples were added to 10 µl genomic DNA digestion mix including 1 µl 100,000‐fold diluted ERCC RNA Spike‐In Mix I and incubated under the same conditions as the oocyte samples. After genomic DNA digestion, 6 µl of the products was dispensed into 96‐well PCR plates and 9 µl RNase‐free water was added to adjust the RNA amount to the oocyte samples, and 1 µl of the diluted products was re‐dispensed into new 96‐well PCR plates. The samples were added to 2 µl component‐adjusting RT‐RamDA mix (4% RealTime ready Cell Lysis Buffer, 0.12% NP40, 1.45× PrimeScript Buffer, 0.16 U DNase I Amplification Grade, 0.6 pmol oligo(dT)18, 8 pmol 1st‐NSRs 100 ng T4 gene 32 protein and 1.5× PrimeScript enzyme mix in RNase‐free water). The samples were incubated under the same conditions as the oocyte samples. After RT‐RamDA, the samples were added to 2 µl second‐strand synthesis mix [2.5× NEB buffer 2 (New England Biolabs), 0.625 mM each dNTP Mixture (TaKaRa), 40 pmol 2nd‐NSRs and 0.75 U Klenow Fragment (3’‐5’ exo‐) (New England Biolabs) in RNase‐free water] and incubated at 16℃ for 60 min, then at 70℃ for 10 min. The second stranded cDNAs were purified using 15 μl AMPure XP beads (Beckman Coulter) and a handmade 96‐well magnetic stand for low volumes. The double‐stranded cDNAs were directly eluted with 3.75 μl 1.33 × diluted Tagment DNA Buffer (Illumina) and mixed well using a vortex mixer and pipetting. Sequencing libraries were generated using the Tn5 tagmentation‐based method with 1/4 volumes of the Nextera XT DNA Library Preparation Kit (Illumina) according to the manufacturer's protocol. Fourteen cycles of PCR were applied for the library DNA. After PCR, sequencing library DNA was purified using 1.2× the volume of AMPure XP beads and eluted into 24 μl TE buffer. RamDA‐seq libraries were quantified and evaluated using a MultiNA DNA‐12000 kit (Shimadzu). The sequencing was performed with Illumina NextSeq 500 High Output v2 (75 cycles; single‐end reads), and 74 individual oocytes and 72 cumulus cell samples were sequenced. Additionally, as a reproducibility experiment, 127 individual oocytes and 127 cumulus cell samples were prepared and sequenced in a same manner.

### 4.4 Sequence data analysis

Hisat2 v2.0.5 (Kim et al., 2015) was used to map the reads to the mouse genome (GRCm38) after trimming adaptor sequences and low‐quality bases using Fastq‐mcf v1.04.807 (Aronesty, 2011). The resulting binary alignment/map (BAM) files were sorted using samtools (Li et al., 2009), and final mapping qualities were assessed using RSeQC v2.6.4 (Wang et al., 2012). The featureCounts v1.5.1 tool of the Subread package (Liao et al., 2013) was used to generate counts of reads multi‐mapped to annotated genes using the GENCODE (vM15) annotation gtf file. We evaluated the quality control metrics to filter out low‐quality samples but our data did not contain a low‐quality sample. We manually removed one pair of samples because following data analysis indicated that the pair did not include oocytes. Using the remaining data derived from 73 individual oocytes and 71 cumulus cell samples, we performed downstream analysis.

Based on the Log_10_ transformed RPKM values of the expression, we visualized *t*‐SNE plots using the Rtsne package in R (Krijthe, 2015). PCA was also conducted using R (R Core Team, 2020).

DEGs (false discovery rate, FDR <0.05) were identified with the edgeR package (Robinson et al., 2009) in R, which normalizes library sizes with the trimmed mean of M values (TMM) method, using the dataset of genes expressed in at least 5 samples. Aging‐ or CR‐associated GO were identified using DAVID v6.8 (GO term Biological Process Direct) (Huang et al., 2008).

To identify potential key transcriptional regulators, we performed regulatory network analysis with a GENIE3 package, a random forest method that computes the links between each gene and all other genes (Huynh‐Thu et al., 2010). We used raw read counts of aging‐ or dietary‐associated DEGs (>0.5 log_2_‐fold coverage, FDR <0.05) as input. Only the transcriptional regulator‐target connected with above thresholds (cumulus: 0.030, oocyte: 0.015) were retained and used for the network analysis. The resulting networks were visualized using CytoScape 3.8 (Shannon et al., 2003).

### 4.5 Immunostaining

Oocytes that underwent nuclear envelope breakdown (NEBD) within 60 min after the induction of meiotic resumption were collected after 14–18hr after NEBD. The oocytes were fixed with a fixation buffer (1.6% paraformaldehyde in 100 mM PIPES (pH7.0), 1 mM MgCl2, 0.1% Triton‐X100) at room temperature for 30 min. After washing 4 times, the oocytes were incubated in PBT (PBS +0.1% Triton‐X100) at 4°C overnight. The oocytes were blocked with 3% BSA‐PBT at room temperature for 1 h and then incubated with human anti‐centromere antibodies (ACA, Antibodies Incorporated, 15–234) at 1:200 in 3% BSA‐PBT at 4°C overnight. The oocytes were washed four times in 3% BSA‐PBT and incubated with secondary antibodies in 3% BSA‐PBT at room temperature for 2 h. The secondary antibody used was Alexa Fluor 488 goat anti‐rabbit IgG (H+L) highly cross‐adsorbed or anti‐human IgG (H+L) cross‐adsorbed (Molecular Probes). DNA was counterstained with 5 µg/ml Hoechst33342 (Invitrogen). For imaging, a customized Zeiss LSM780 confocal microscope equipped with a 40×C‐Apochromat 1.2NA water immersion objective lens (Carl Zeiss) was used. We recorded z‐confocal sections (every 0.5 μm) of 512 × 512 pixel xy images to capture all chromosomes.

For cohesin immunostaining, we used a protocol modified from a previous study (Lee et al., 2011). Fully grown oocytes were incubated in IBMX‐free M2 medium for 5 h at 37°C. Oocytes at metaphase I were microinjected with 4 pl purified Rec8 antibody (0.4 mg/ml) (Ding et al., 2018) and incubated for 8 min at 37°C before fixation.

### 4.6 Image analysis

Fiji (https://fiji.sc/) was used to quantify fluorescent signals. To define regions of interest (ROIs) for Rec8 signals, a threshold value for segmentation was defined by the Otsu algorithm with z‐projected, Gaussian (sigma 2)‐blurred images around chromosomes. The threshold value was used for image segmentation on individual z‐slices. The integrated signal intensities on ROIs of all z‐slices were used to calculate the mean intensity of Rec8 signals on chromosomes. Cytoplasmic mean intensity was measured by manually selecting a representative region. The mean intensity of Rec8 on chromosomes was subtracted with the cytoplasmic intensity. The procedure of image analysis was automated with an in‐house macro in Fiji.

For counting the number of kinetochores, the positions and number of kinetochores were manually determined in 3D‐reconstructed images in Imaris (Bitplane).

## CONFLICT OF INTEREST

The authors confirm that they have no conflict of interest.

## AUTHOR CONTRIBUTIONS

T.M. performed data analysis and interpreted the data. N.T. performed the CR experiments, cohesin quantification, and sample preparation. T.H., M.Y., and M.U. performed RamDA‐seq and data analysis. M.M. and Y.I. performed sample preparation. H.H., I.N., and T.S.K. designed, conceptualized and supervised the project, interpreted the data and wrote the manuscript.

## Supporting information

Fig S1‐S4

## Data Availability

The RamDA‐seq data of mouse oocytes and cumulus cells were deposited into Gene Expression Omnibus (GEO) repository and are accessible through GEO accession number GSE159281.
