# Supplementary material for: Single‐oocyte transcriptome analysis reveals aging‐associated effects influenced by life stage and calorie restriction
Source: Aging Cell. 2021 Jul 10;20(8):e13428. doi: 10.1111/acel.13428 (PMC8373347; doi:10.1111/acel.13428)
Supplement: Supplementary file 1 — Fig S1‐S4 [file ACEL-20-e13428-s001.pdf]

Supporting Information

Single-oocyte transcriptome analysis reveals aging-associated effects influenced by life stage and calorie restriction

Correspondence to: tomoya.kitajima@riken.jp (T.S.K.)

Figure S1

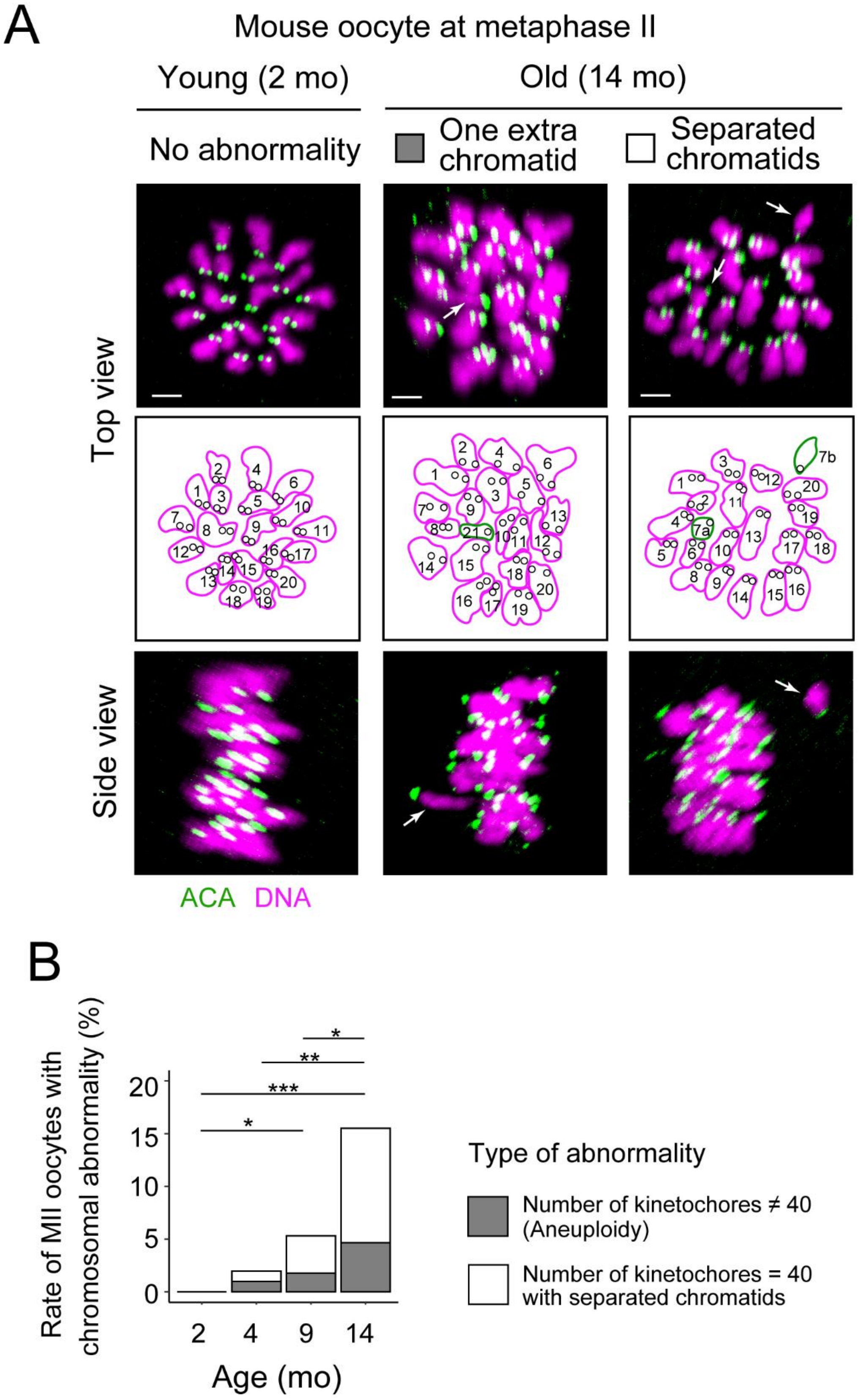
A
Mouse oocyte at metaphase II
Young (2 mo)
Old (14 mo)
No abnormality
One extra chromatid
Separated chromatids
Top view
Side view
ACA DNA
B
Rate of MII oocytes with chromosomal abnormality (%)
Age (mo)
2
4
9
14
Type of abnormality
Number of kinetochores ≠ 40 (Aneuploidy)
Number of kinetochores = 40 with separated chromatids

Figure S1 Aging-associated increase in chromosome abnormality.

(A) Whole-mount 3D imaging of oocytes for chromosome and kinetochore counting. Oocytes at metaphase II were immunostained for kinetochores (ACA, green) and DNA (Hoechst33342, magenta). The positions and number of kinetochores were determined in 3D-reconstructed images. Arrows indicate one extra chromatid and separated chromatids. Projection views from the top and side of the metaphase plate are shown. For visualization, signals are interpolated in z. Scale bar, 2 μm.

(B) Aging-associated increase in chromosome segregation errors. Images shown in (A) were used for detecting abnormalities in chromosome and kinetochore numbers (n = 124, 102, 113, 129 oocytes). Note that "Number of kinetochores ≠ 40" (aneuploidy) is due to chromosome segregation errors (nondisjunction or unbalanced predivision) at meiosis I, and that "Number of kinetochores = 40 with separated chromatids" is due to chromosome segregation errors (balanced predivision) at meiosis I or premature separation of sister chromatids at metaphase II. *p<0.05, **p<0.01, and ***p<0.001 (Fisher's exact test with holm correction).

Figure S2

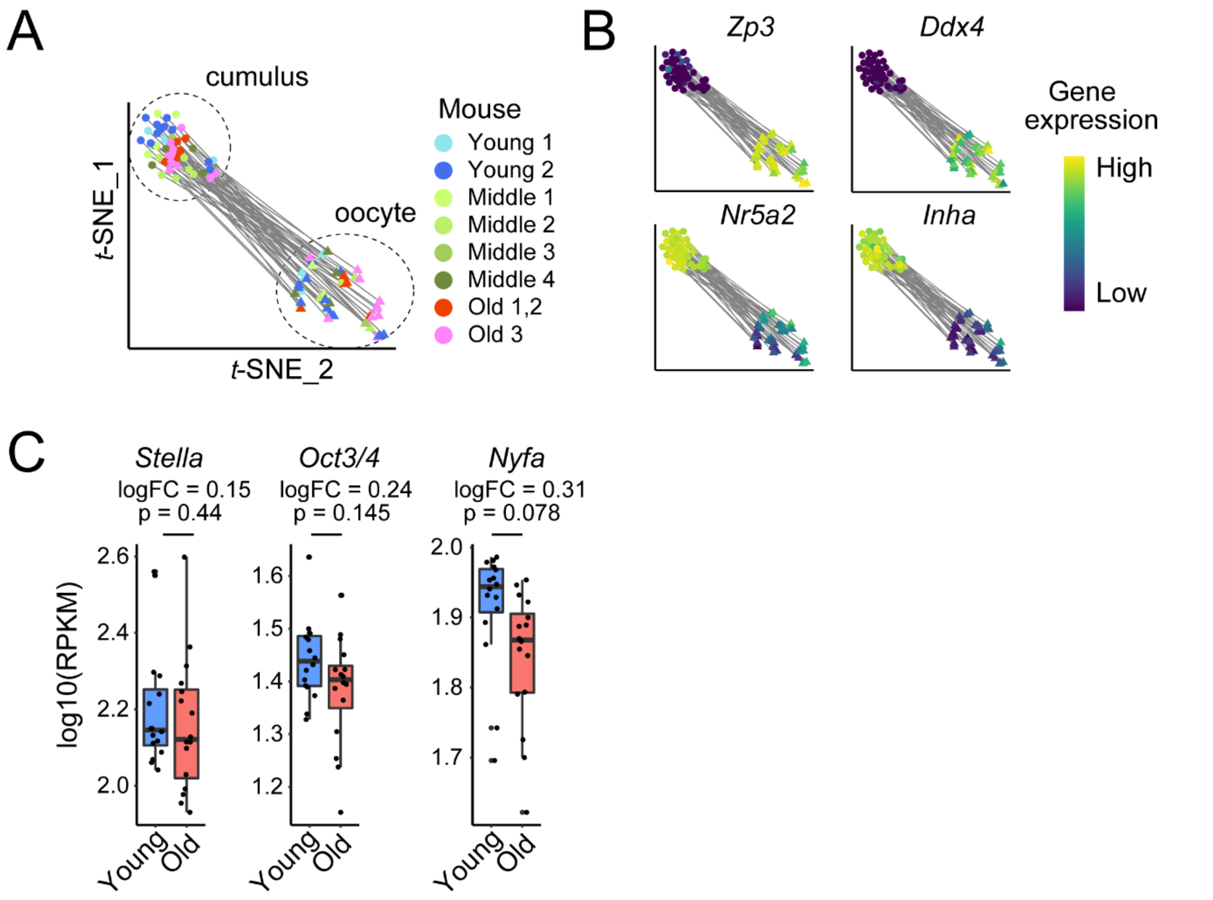

Figure S2 Transcriptome profiling of single oocytes and their surrounding cumulus cells from mice at different life stages.

(A) *t*-SNE plot showing the distribution of single oocytes and their surrounding cumulus cells. Circles and triangles represent cumulus cells and oocytes, respectively. The pairs of oocytes and their surrounding cumulus cells are connected with a line. Colors indicate individual mice.

(B) Expression of markers for oocytes and cumulus cells. *Zp3* and *Ddx4* are oocyte markers. *Nr5a2* and *Inha* are cumulus cell markers.

(C) Expression of markers for fully grown oocytes. Levels of *Stella*, *Oct3/4* and *Nyfa* are shown.

Figure S3

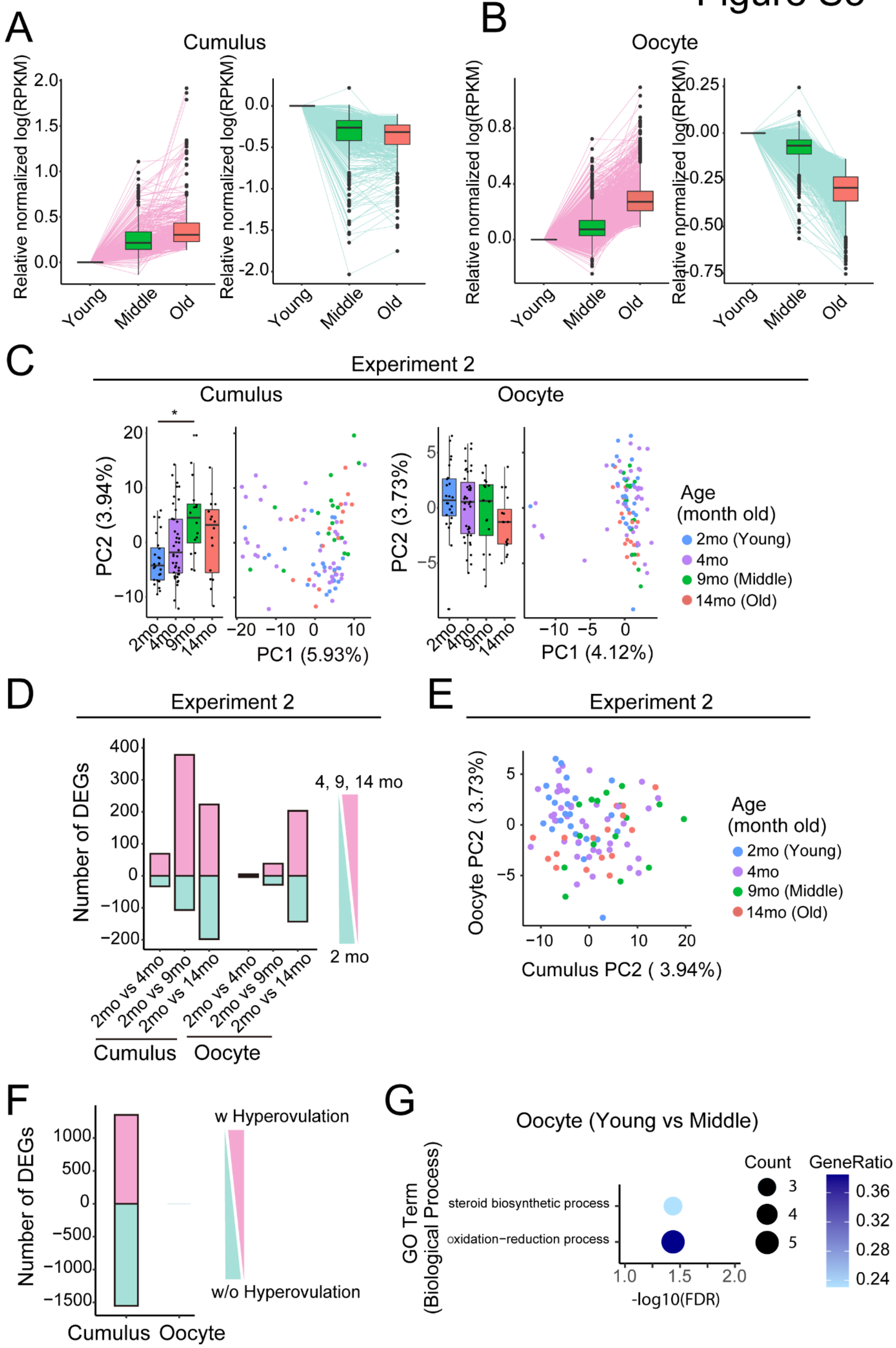
A
Cumulus
Relative normalized log(RPKM)
Young
Middle
Old
B
Oocyte
C
Experiment 2
Cumulus
Oocyte
PC2 (3.94%)
PC1 (5.93%)
PC2 (3.73%)
PC1 (4.12%)
Age (month old)
2mo (Young)
4mo
9mo (Middle)
14mo (Old)
D
Experiment 2
Number of DEGs
4, 9, 14 mo
2 mo
2mo vs 4mo
2mo vs 9mo
2mo vs 14mo
Cumulus
Oocyte
E
Experiment 2
Oocyte PC2 (3.73%)
Cumulus PC2 (3.94%)
Age (month old)
2mo (Young)
4mo
9mo (Middle)
14mo (Old)
F
Number of DEGs
w Hyperovulation
w/o Hyperovulation
Cumulus
Oocyte
G
Oocyte (Young vs Middle)
GO Term (Biological Process)
steroid biosynthetic process
oxidation−reduction process
-log10(FDR)
Count
GeneRatio

Figure S3 Distinct characteristics of aging-associated genes between oocytes and cumulus cells.

(A, B) Line plots showing expression pattern of up-regulated (left) or down-regulated (right) DEGs between young, middle and old mice. (A) cumulus cells. (B) oocytes.

(C) PCA plot for the experiment 2 using all expressed genes showing an aging-associated distribution. (left) cumulus cells and (right) oocytes. The number of oocyte-cumulus complex from 2 months, 4 months, 9 months, and 14 months old mice were 24, 39, 16, and 16, respectively.

(D) Histogram showing the number of up-regulated or down-regulated DEGs between 2 months old and other age groups in the experiment 2.

(E) Plot showing the relationship between eigen values of aging-associated PC axes from oocytes and that from their corresponding cumulus cells in the experiment 2.

(F) Histogram showing the number of up-regulated or down-regulated DEGs in oocytes and cumulus cells from 2-months-old mice with hyperovulation (n = 24) and without hyperovulation (n = 32).

(G) Representative GO terms of down-regulated enrichment between young and middle oocytes.

Figure S4

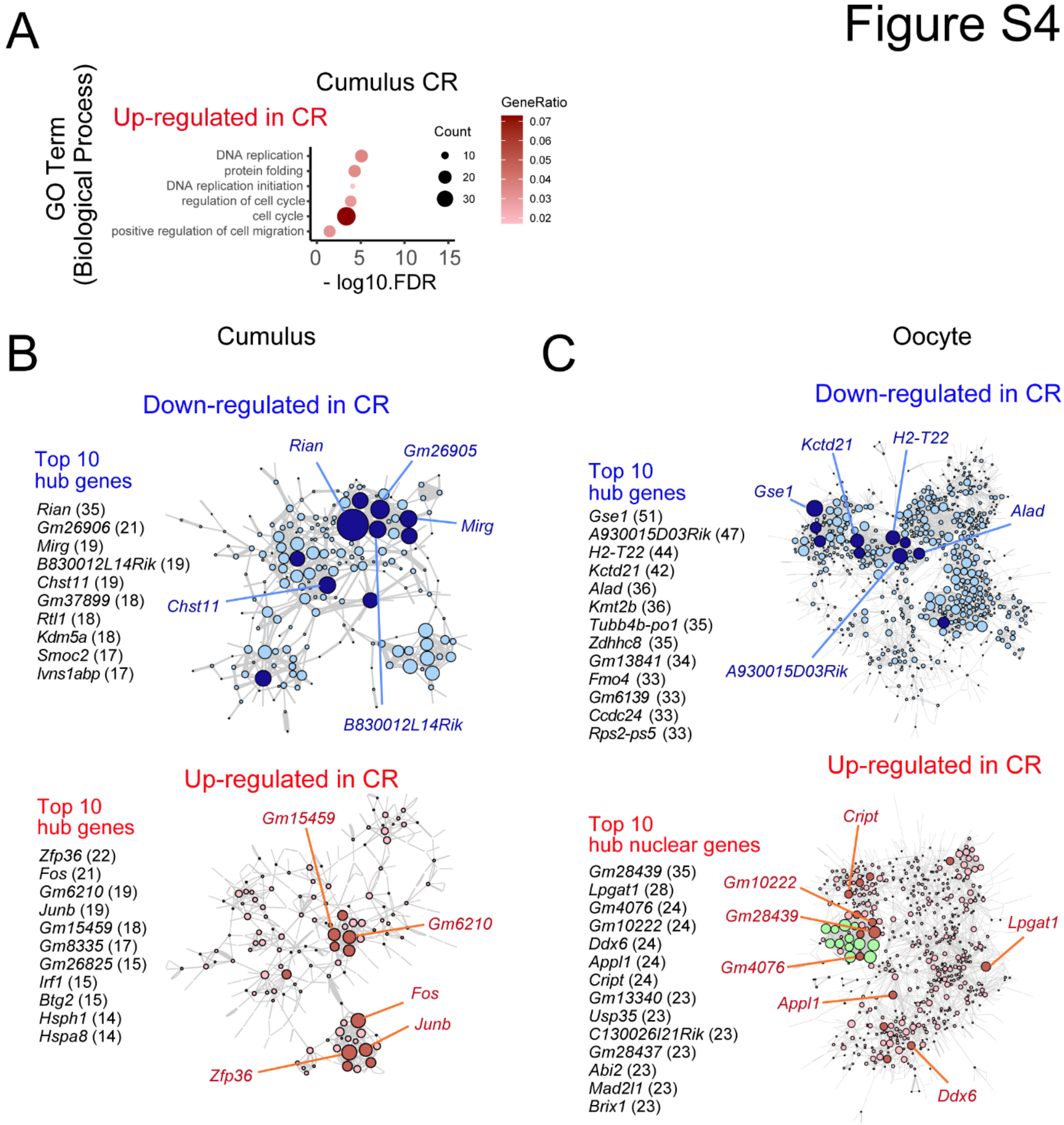

Figure S4 CR effects.

(A) Representative GO terms of up-regulated enrichment between AL-middle and CR-middle cumulus cells.

(B, C) Regulatory networks visualizing potential key transcriptional regulators (B) in cumulus cells and (C) in oocytes associated with CR. Down-regulated and up-regulated top 10 nodes were colored in dark blue and dark red, respectively. Top 10 hub nuclear genes are shown. Green spots indicate mitochondrial genes. The threshold used for regulator-target connection in oocytes was lower than in cumulus cells.
